# Re-framing eco-distress for self-efficacy and resilience building

**DOI:** 10.1371/journal.pmen.0000563

**Published:** 2026-02-20

**Authors:** Bill Sheate

**Affiliations:** 1 Bloomsbury Therapy Centre, London, United Kingdom; 2 Centre for Environmental Policy, Imperial College London, London, United Kingdom; PLOS: Public Library of Science, UNITED KINGDOM OF GREAT BRITAIN AND NORTHERN IRELAND

## Introduction

Research in the field of eco-anxiety, eco-distress and ‘climate emotions’ has exploded in recent years [1] as has public awareness. The evidence base for the negative impacts of climate and environmental change on public mental health continues to increase steadily [2]. At the same time, resilience among young people in particular, and working age people more generally, has shown a steady decline through the Covid-19 pandemic and beyond due to multiple factors (e.g., educational pressures, social media, cost of living crisis, limited access to mental health services etc); the percentage of people in the UK, for example, with poor mental health has doubled since 2010 [3,4], even greater in the US [5]. In this light, the global climate crisis is one – but not the only one - of several major concurrent social changes young people and others are having to deal with. Given this, an important question is whether eco-distress is so different from other forms of distress that it requires a completely different approach, as some argue [6,7]? This paper suggests not.

## Framing – the influence of research in shaping the narrative around eco-distress

Chiolero and others caution as to the way in which some eco-distress research is being framed [8] and its potential impact upon awareness, perception and prevalence of eco-distress itself [9]. We can also see how drivers of research like funding and the philosophical values of researchers themselves can shape research priorities [8,10]. It is not surprising that much of the literature in this area has been framed with a strong environmental, social justice and climate action perspective and with rather less on a therapeutic imperative. Indeed, that is often deliberate as a desire to normalise rather than pathologize the various forms of eco-distress. But the framing has consequences in that it may contribute to the very thing being studied [8,9]: greater awareness brings greater self-identification and labelling and for some, this can contribute to unhelpful coping strategies [11,12]. While eco-distress may not be a psychological disorder it does not mean it is not a mental health problem [13] and we know that those vulnerable to anxiety and depression are more likely to experience eco-distress [2,12,14].

Psychosocial responses to climate and environmental change, in the clinic room, are not so different to responses to other triggers of anxiety, sadness or anger. A global or existential dimension is not so unusual as a cause of mental ill health; similar can be seen in relation to other natural disasters, pandemics, conflict, geopolitics, and more day-to-day issues. While the context may be different, the core underlying maladaptive processes remain essentially the same: overthinking (worry, rumination), experiential avoidance, focus of (or ‘stuck’) attention, intolerance of uncertainty, social isolation, procrastination, and doomscrolling [11,12]; all can be targeted with the client and context (e.g., climate crisis) in mind, if the therapist appreciates the environmental context. We know that greater resilience is associated with better mental health outcomes [14–16], so building self-efficacy and long-term resilience can allow us to address multiple factors challenging public mental health.

## Re-framing eco-distress

The recent emphasis in the literature on ‘climate (or eco-) emotions’ [6,7] implies a debateable uniqueness of these emotional responses; included in this taxonomy are often a number of ‘emotions’ that arguably aren’t really emotions at all, but that more closely align to cognitions, or even processes (like ‘grief’); similar criticisms have been levelled at Plutchik’s emotions wheel [17]. While a *‘climate emotions wheel’* might work practically in surveys and workshops, it risks prompting individuals to label themselves and identify with a pre-determined set of ‘emotions’ rather than encourage them to tease apart their own thoughts, feelings and behaviours about climate change themselves. *‘I feel helpless’*; *‘It feels hopeless’*, for example, are not really emotions at all even though individuals may describe them as how they *feel* – they are thoughts (cognitions), i.e., ‘I feel the way I feel (sad, angry, anxious) when I *believe* there is nothing *I* can do or when I believe no one is ever going to do anything.’

Paradoxically, a focus on a defined list of (‘off-the-shelf’) *climate* ‘emotions’ may reduce self-efficacy more generally if it discourages personal development of wider emotional literacy and regulation. Rather than a focus on ‘climate emotions’ a more helpful framing would be around building self-efficacy and resilience generally to factors contributing to declining public mental health, including the climate crisis.

## Building personal self-efficacy and resilience

To build resilience means having psychological flexibility, to be able to deal with life’s ups and downs through appropriate responses and emotional regulation [14]. We can see this described, for example, in Acceptance and Commitment Therapy (ACT) and other individualised, mindfulness-based transdiagnostic cognitive behavioural therapies [18]. Central to this is an openness to present moment awareness, acceptance (of feelings as a normal part of human experience), defusion of thoughts (letting go of unhelpful negative thoughts) and, as an observer of your thoughts and feelings, being able to take committed action (in the present moment) in service of (multiple) values – the domains of life that are important to the individual. In eco-distress it is this shift in perspective that can help re-connect people to other values that are important to them as well as the environment. Yet one increasingly common (but maladaptive) behavioural response by young people is experiential avoidance of exposure to low level anxiety, for example through excessive headphone use [19], leading to greater isolation, less connectedness and low levels of experience of the present moment, i.e., decreased resilience generally.

## Practical implications for public mental health

We want people to be resilient – to climate change but also other things that life can throw at them; to be better able to manage and help themselves. Rather than seeing psychosocial responses to climate change as unique requiring separate resourcing or action we may be missing out on helping people to help themselves more generally *and* around climate issues. The common crisis is in the decline in resilience especially among young people to anxiety and depression, the rise in avoidant coping strategies (often through technological innovation) and lack of access to appropriate mental health support. The teaching of basic CBT and mindfulness principles (psycho-education) and self-help skills in schools, colleges and universities, and greater awareness of how to deal with key stress factors such as phone screentime, doomscrolling, and avoidance of exposure to low level normal anxious thoughts and feelings would go a long way to equip young people and society with greater agency, self-efficacy, and resilience to stress in its many manifestations.

Governments should address climate change mitigation and adaptation as a matter of urgency, but that alone will not stop young people from experiencing stress, anxiety, and depression in relation to climate change or in other contexts. If you give them the skills to better manage stress, anxiety and depression you give them the skills to manage eco-distress and act in ways that matter to them personally. From a public mental health perspective, we need to see eco-distress as a symptom of the wider mental health crisis rather than as a specific mental health crisis of its own.
